# Characterization of Canine Peyer’s Patches by Multidimensional Analysis: Insights from Immunofluorescence, Flow Cytometry, and Single-Cell RNA Sequencing

**DOI:** 10.4049/immunohorizons.2300091

**Published:** 2023-11-28

**Authors:** Beatriz Miguelena Chamorro, Sodiq Ayobami Hameed, Marianne Dechelette, Jean-Baptiste Claude, Lauriane Piney, Ludivine Chapat, Gokul Swaminathan, Hervé Poulet, Stéphanie Longet, Karelle De Luca, Egbert Mundt, Stéphane Paul

**Affiliations:** * Centre International de Recherche en Infectiologie, Team GIMAP (Saint-Etienne), Université Claude Bernard Lyon 1, Inserm, U1111, CNRS, UMR5308, ENS Lyon, UJM, F69007 Lyon, France; † Global Innovation, Boehringer Ingelheim, Saint-Priest, France; ‡ International Center for Infectiology Research, INSERM 1408 Vaccinology, Saint-Etienne, France

## Abstract

The oral route is effective and convenient for vaccine administration to stimulate a protective immune response. GALT plays a crucial role in mucosal immune responses, with Peyer’s patches (PPs) serving as the primary site of induction. A comprehensive understanding of the structures and functions of these structures is crucial for enhancing vaccination strategies and comprehending disease mechanisms; nonetheless, our current knowledge of these structures in dogs remains incomplete. We performed immunofluorescence and flow cytometry studies on canine PPs to identify cell populations and structures. We also performed single-cell RNA sequencing (scRNA-seq) to investigate the immune cell subpopulations present in PPs at steady state in dogs. We generated and validated an Ab specifically targeting canine M cells, which will be a valuable tool for elucidating Ag trafficking into the GALT of dogs. Our findings will pave the way for future studies of canine mucosal immune responses to oral vaccination and enteropathies. Moreover, they add to the growing body of knowledge in canine immunology, further expanding our understanding of the complex immune system of dogs.

## Introduction

Oral vaccines are promising tools for combating pathogens in both human and veterinary medicine, as they are easy to administer and can induce mucosal immune responses (1). The possibility of using the mucosal route of immunization in dogs has excited considerable interest. For instance, oral rabies vaccines successfully protect wild animals and consequently humans (2). This route has also been licensed for vaccines against respiratory pathogens, such as *Bordetella bronchiseptica*, resulting in up to 13 mo of protection in dogs (3), although the mechanisms behind the memory responses are not yet deciphered.

Following oral vaccine administration, the oral mucosa and GALT induce a strong local mucosal immune response. The GALT contains inductive sites, including lymphoid follicles known as Peyer’s patches (PPs), which are distributed along the length of the small intestine wall, and mesenteric lymph nodes (MLNs) (4). PPs contain follicular B cell areas housing germinal centers (GCs), whereas the adjacent interfollicular areas contain diverse cell types, including T cells (5). Somatic hypermutation and Ab affinity maturation occur in the GCs (6). These structures are lined with the follicle-associated epithelium (FAE), which contains M cells, being specialized cells responsible for luminal Ag sampling (7). The subepithelial dome (SED), a region containing abundant dendritic cells (DCs), underlies the FAE. The DCs in this region carry Ags to naive T cells in the PPs or to the draining MLNs via the lymph vessels (4). The MLNs are also important inductive sites, with areas containing B cell follicles, GCs, and T cells, in which the immune responses of PPs are initiated and amplified and the exit of activated B and T cells from the intestinal mucosa is facilitated (4). CD103^+^ migratory DCs play a significant role in imprinting gut-homing α_4_β_7_^high^ CCR9^+^ CD4 and CD8 effector T cells (8). Activated cells from the inductive sites migrate, guided by homing receptors, to the lamina propria (LP) or other effector sites, where they perform their functions (4).

The successful development of effective oral vaccines against pathogens therefore depends on improvements in our understanding of the canine GALT. For canine oral vaccines, our understanding is currently limited by the scarcity of data relating to the mucosal immune system and, consequently, mucosal immune responses. Several studies on the canine mucosal immune system have addressed important aspects, such as the induction of mucosal IgA in the nasal cavity (9) and local cellular responses (10) following mucosal immunization. Others have provided valuable insights into the canine intestine, particularly in the context of enteropathies (11, 12). However, despite these efforts, our understanding of the local tissue-specific responses to mucosal vaccines in dogs remains fragmented. One specific area requiring further exploration is the cell composition and structure of PPs. Additional studies are required, building on the pioneering work of almost three decades ago providing the first glimpse into the world of canine PPs (13). These investigations revealed the widespread distribution of these structures along the entire length of the intestine, with marked differences between the canine PPs of the duodenum and jejunum and those of the ileum (13).

“Microfold” or M cells, specialized cells with a key role in mucosal response induction in the GALT and the generation of IgA responses (14), have not been thoroughly characterized in dogs. Given their role in the GALT, various strategies have been developed for targeting these cells for mucosal vaccination, in both humans (15) and veterinary medicine (16). Vaccination with a molecule targeting M cells in mice, but not guinea pigs, has been shown to improve IgA and cellular responses in mice (16). However, the presence and frequency of these cells in dogs will need to be evaluated to determine the feasibility of this strategy in this species. Heterogeneity has been observed between species, with M cells covering ∼5–10% of the FAE in humans and mice, but almost 50% in rabbits (17).

We therefore performed a multidimensional analysis of canine PPs. We first performed immunofluorescence analyses to characterize the cell populations present and their distribution, encompassing immune cells as well as specialized epithelial cells. We also studied intestinal immune cell markers by flow cytometry. We performed single-cell RNA sequencing (scRNA-seq) to investigate the subpopulations of cells present at mucosal induction sites. This technique identified several B and T cell subpopulations as the major components of these tissues and enabled us to develop an atlas of the main cell populations. Our findings have significant implications for further research targeting the GALT, encompassing canine oral vaccines, canine enteropathies, and the use of dogs as models of human diseases. By focusing on previously unexplored aspects of the mucosal immune system in dogs, this study extends our basic knowledge of canine immunology. Tools for studying immunology in dogs are less well developed than those for humans or rodents. The validation and extension of these findings is therefore of great importance for the canine research community and for mucosal immunologists.

## Materials and Methods

### Ethics statement

All of the animal studies were reviewed and approved by the Institutional Animal Care and Use Committee, which ensured that all experiments conformed with the relevant regulatory standards (directive EU2010/63) and the Corporate Policy on Animal Welfare (029-DCPOL-001).

### Animals

The intestines used in this study were obtained from healthy dogs housed in the animal facilities of Boehringer Ingelheim Animal Health. These dogs, which previously participated in other research projects, provided the intestines used in our study at the conclusion of those unrelated projects to support our research. In particular, the dogs involved were all healthy females aged between 6 mo and 1 y, and the previous procedures they underwent did not specifically involve or directly affect their intestinal health. Specifically, the duodenum and jejunum of two dogs were used for immunofluorescence analyses. The intestines of two additional dogs were used for flow cytometry, and the intestines of four dogs were used for the scRNA-seq experiments. The intestines were transferred from the animal facilities at 4°C, in HBSS medium without Ca^2+^ and Mg^2+^ (Life Technologies, 14175053) and supplemented with gentamicin (Sigma-Aldrich, G1397), penicillin/streptomycin (Life Technologies, 15070063), and HEPES (Sigma-Aldrich, H0887).

### Cell isolation from PPs for flow cytometry and scRNA-seq

The PPs were harvested by placing the duodenum and jejunum on paper towels moistened with calcium- and magnesium-free (CMF) buffer (10 mM HEPES, 2% FBS [Life Technologies, 1043-000183] in HBSS without Ca^2+^/Mg^2+^), and the oval thumbprint-sized PPs were carefully harvested. The PPs were then transferred to petri dishes with cold CMF buffer (4°C) to remove debris. Intestinal epithelial cells were then separated by cutting the PPs into small pieces (∼2 cm) and transferring to a tube with 40 ml of prewashing buffer solution (CMF, 2 mM DTT [Sigma-Aldrich, 43816], 0.5 mM EDTA), which was then incubated at 37°C with 5% CO_2_ for 30 min with continuous stirring. The tubes were vortexed vigorously, and the supernatants were discarded, followed by a 20-min incubation period and subsequent vortexing. An additional incubation was carried out at 37°C for 30 min in 10 ml of 100 U/ml collagenase solution containing RPMI 1640 medium (Life Technologies, 52400041), 10% FBS, gentamicin, 100 U/ml collagenase type I (Life Technologies, 17100-017), and 25 U/ml DNase I (Roche, 04716728001), followed by a 15-s vortexing of the tube. The tissues were transferred into two gentleMACS C tubes (Miltenyi Biotec, 130-093-237), each containing 10 ml of prewarmed gut medium (5% FBS, RPMI 1640, gentamicin, at 37°C). They were then dissociated by running the C_01 program of the gentleMACS dissociator (Miltenyi Biotec, 130-096-427) twice. The dissociated cells were subsequently filtered through a 70-µm pore size cell strainer (Corning, CLS431751-50EA) and centrifuged at 4°C and 500 × *g* for 10 min after supplementation with gut medium. The cells were resuspended in 40% Percoll density gradient (Cytiva, 17-0891-02) and layered onto 70% Percoll. The gradient was centrifuged for 30 min at 900 × *g* with the centrifuge brake set at level 3. The leukocytes were collected at the 40–70% Percoll interphase. After washing and centrifugation, the cell pellet was resuspended in 2 ml of sterile complete RPMI 1640. Cell viability was assessed using trypan blue staining under a light microscope, using a plastic KOVA slide device.

### Production of human combinatorial Ab libraries Abs targeting canine GP2

For the generation of mAbs against the canine GP2 protein, a small part of the canine GP2 ectodomain (GenBank accession no. NM_001003371) from aa 32 to 189 was expressed in HEK293 cells having a GGGSG amino acid sequence as spacer and a 9× His tag sequence encoded at its N terminus for subsequent purification (Trenzyme, Konstanz, Germany). The purified protein (>99% purity) was transferred to Bio-Rad AbD Serotec (Puchheim, Germany) for the generation of mAbs using the human combinatorial Ab libraries Abs (HuCAL) technology, as previously described (18). In summary, the HuCAL Platinum phage library underwent three rounds of panning to isolate Abs specific to the purified part of the ectodomain of canine GP2. Subsequently, the obtained library was subcloned into a bacterial expression vector to generate several Abs in the Fab format. Following further screening against canine GP2, 17 clones were sequenced, expressed, and purified by affinity chromatography and converted into human IgG1-FcSpyCatcher–like His-tagged Abs.

### Human, canine, and murine GP2 cell expression

To study the commercial Abs against GP2 of different species, three different plasmids (pcDNA 3.1) encoding for the full-length sequence of human (GenBank accession no. NM_001007240), canine (GenBank accession no. NM_001003371), and murine (GenBank accession no. XM_006508121.2GP2) GP2 and for a FLAG tag amino acid sequence (DYKDDDDK) at its C terminus were used for transfection. The genes were synthesized and provided cloned into the eukaryotic expression plasmid pcDNA 3.1 (Azenta Life Science, Leipzig, Germany). For the preparation of the plasmids, Luria–Bertani (LB) broth ampicillin agar plates were prepared by adding sterile water to an imMedia amp agar pouch (Invitrogen, 45-0034). A tube of competent cells (NEB 5-alpha competent *Escherichia coli*, New England Biolabs, C2988H) was thawed on ice, and DNA plasmids were introduced separately into the tubes with competent cells. The tubes were incubated on ice for 30 min, heat-shocked for 30 s at 42°C, and then placed on ice again for 5 min. Next, SOC outgrowth medium (New England Biolabs, B9035S) was added to the tubes, followed by incubation at 37°C for 1 h under rotation. The transformed cells were spread selectively on LB amp plates (prewarmed to 37°C) and incubated overnight at 37°C. The next day, LB broth (Life Technologies, 10855-001) ampicillin (100 mg/ml) medium was prepared, and harvested bacterial colonies were incubated at 37°C and 225 rpm overnight. Plasmid DNA was prepared using the QIAprep spin miniprep kit (Qiagen, 27104) and confirmed by restriction enzyme digest and agarose gel analysis. Subsequently, a glycerol stock was prepared by mixing bacterial culture with glycerol and storing stored it at −70°C. Plasmids for transfection were purified using a HiSpeed plasmid midi kit (Qiagen, 12643) following the manufacturer’s instructions. Briefly, the bacterial pellet was resuspended, and lysate was filtered into a HiSpeed tip, eluted, precipitated, washed, and finally eluted again in RNase DNase-free water. The identity of the batch was confirmed first by restriction enzyme digest analysis followed by sequencing. The mixture underwent gel electrophoresis for DNA analysis, and the DNA concentration was measured using a P200 Picodrop spectrophotometer (Picodrop, Hinxton, U.K.).

### Cell transfection and immunofluorescence assay for GP2 evaluation in BHK-21 cells

We assessed the expression of FLAG-tagged GP2 proteins from different species (human, mouse, and dog) by transfecting BHK-21 cells with the corresponding plasmids in the presence of Lipofectamine 3000 (Invitrogen, 3000015). The day before transfection, BHK-21 cells were plated in T75 flasks, in Glasgow MEM (Life Technologies, 1045-001089) supplemented with 10% FBS and 0.2% penicillin/streptomycin. The cells were detached, counted with an Invitrogen Countess automated cell counter, and plated at a density of 25 × 10^3^ cells per well in the same medium. Next day, the cells were transfected with plasmids encoding either the human, mouse, or canine GP2-FLAG proteins. The transfection was performed using Lipofectamine 3000 following the manufacturer’s instruction using ∼1 µg of plasmid DNA per well. Forty-eight hours after transfection, the medium was removed and cells rinsed with 4°C cold PBS (Life Technologies, 70011044) and subsequently treated with absolute ethanol at −20°C. The ethanol was removed and cells were dried under the laminar flow and stored at 4°C wrapped in aluminum foil. For immunofluorescence, cells were rehydrated with PBS at room temperature. To minimize background fluorescence, cells were incubated with a filtered blocking buffer containing Triton X-100 (Sigma-Aldrich, T8787), goat serum (Vector Laboratories, S-1000), and albumin (Roth, 3737.3) in 1× PBS without Ca^2+^/Mg^2+^. Ab dilutions and incubations were performed in the blocking buffer, and cells were rinsed with PBS between different Abs. To validate the expression of the proteins a rabbit anti-FLAG Ab (Sigma-Aldrich, F7425) and goat anti-rabbit Alexa Fluor Plus IgG (H+L) 488 (Invitrogen, A32731) were used. After validating the expression of the proteins, the following Abs were used in the transfected cells: mouse anti-human GP2 (LS Bio, LS-C761676), rat anti-mouse GP2 (MBL, D278-5), and human anti-canine GP2 (produced for this study by Bio-Rad). The secondary Abs included goat anti-human IgG FITC (Invitrogen, 31531), goat anti-rat IgG FITC (Bio-Rad, 305002), and goat anti-mouse IgG FITC (Bio-Rad, 103001F). Hoechst stain (Invitrogen, 2306347) was used to stain the nuclei of the cells at a final concentration of 1µg/ml. Fluorescence was analyzed using a Zeiss Axiovert (Carl Zeiss, Oberkochen, Germany).

### Tissue preparation and immunofluorescence

Canine PPs were carefully isolated from the duodenum and jejunum and flushed with PBS. Each PP was frozen in OCT embedding matrix (CellPath, 6478.1) and cut into 7-µm sections on a Leica CM3050 S cryostat (Leica Biosystems). The sections were captured on Fisherbrand Superfrost Plus microscope slides (Epredia, J1800AMNZ), which were then washed in PBS to eliminate residual OCT. Depending on the Ab staining requirements, different procedures were employed for the slides. Some slides (CD11c) underwent a 15-min incubation in 95% acetone at −20°C, whereas others were fixed using 4% paraformaldehyde (Sigma-Aldrich, P6148) in 1× PBS. The latter group was slightly permeabilized by two 5-min incubations with 0.05% saponin (Sigma-Aldrich, 47036) in 1× PBS (canine GP2), maintaining saponin levels at 0.005% throughout the remaining steps. Another set of slides was permeabilized by a 1-h incubation with 0.1% Triton X-100 (Sigma-Aldrich, T8787) in 1× PBS (FOXP3) at room temperature, whereas the remaining samples proceeded directly to the next step. All slides were then blocked by incubation with blocking buffer (5% BSA in PBS; Seqens, 1000-70) for 1 h at room temperature. The slides were then incubated overnight at 4°C with 1:100 to 1:200 dilutions of the following Abs in blocking buffer: mouse anti-dog CD3 (Bio-Rad, MCA1774GA), rat anti-dog CD8 (Bio-Rad, MCA1039GA), mouse anti-dog CD21 (Bio-Rad, MCA1781R), mouse anti-dog CD4 (Bio-Rad, MCA1998S), mouse anti-human GP2 (LS Bio, LS-C761676), rat anti-mouse GP2 (MBL, D278-5), human anti-dog GP2 (produced for this study by Bio-Rad), rat anti-bovine/dog FOXP3 FITC (Invitrogen, 11-5773-82), mouse anti-human/dog CD14 (Bio-Rad, MCA1568GA), and mouse anti-dog CD11c (Bio-Rad, MCA1778S). The slides were then washed in PBS and incubated with the secondary Abs, that is, goat anti-rat IgG FITC (Bio-Rad, 305002), goat anti-mouse IgG FITC (Bio-Rad, 103001F), goat anti-mouse IgG RPE (Bio-Rad, 103009), or goat anti-human IgG FITC (Invitrogen, 31531), for 1 h at room temperature. The slides were washed twice in PBS, allowed to dry in air, and were then mounted in FluoroShield with DAPI (Sigma-Aldrich, F6057) and observed under a Nikon Eclipse Ti immunofluorescence microscope.

### Flow cytometry analysis of canine PPs

Flow cytometry analysis was performed to assess cell viability and to identify the population of immune cells in the PPs. The tissue was processed as previously described in this study and 500,000 cells per well were dispensed into deep 96-well plates. The cells were initially washed in FACS buffer (0.5% BSA, 0.02% sodium azide in PBS, filter-sterilized) and centrifuged at 400 × *g* for 5 min at 5°C. The resulting cell pellets were resuspended in 200 µl of LIVE/DEAD fixable yellow dead stain (Invitrogen, L34959) diluted 1:1000 in PBS and incubated for 20 min at 4°C. The cells were then washed twice with 200 µl of FACS buffer and resuspended in 50 µl of FACS buffer containing the Abs for surface staining. We used the following Abs: mouse anti-dog CD3 Alexa Fluor 700 (Bio-Rad, MCA1774A700), rat anti-dog CD4 PE-Cy7 (Bio-Rad, MCA1038PeCy7), rat anti-dog CD8 Pacific Blue (Bio-Rad, MCA1039PB), and mouse anti-dog CD21 Alexa Fluor 647 (Bio-Rad, MCA1781A647). The plates were incubated for 20 min at 5°C in the dark, then washed twice with 200 µl of FACS buffer/well and centrifuged for 5 min at 400 × *g* at 5°C. For intracellular staining, cells were washed as described above and fixed by incubation with 1× FOXP3 Fix/Perm solution from the eBioscience Foxp3/transcription factor staining buffer set (Invitrogen, 00-5523-00) for 30 min in the dark. Samples were washed twice with the permeabilization buffer from the same set and incubated with rat anti-bovine/dog FOXP3 FITC (Invitrogen, 11-5773-82) and rat anti-human/mouse B cell lymphoma 6 (Bcl-6) allophycocyanin (BioLegend, 358506) for 30 min. The cells were washed twice with permeabilization buffer as described above and fixed in 1% paraformaldehyde in 1× PBS. The samples were analyzed with a Cytek Aurora (Cytek Biosciences, Fremont, CA) and FlowJo version 10.8.1 (Becton Dickinson, Franklin Lakes, NJ).

### scRNA-seq library preparation

Cells suspensions were prepared as previously described in this study, and scRNA-seq library preparation was performed with the Chromium Next GEM (gel beads-in-emulsion) single-cell 3′ reagents kits v3.1 (10x Genomics, Pleasanton, CA) (Supplemental Table I). The instructions supplied by the manufacturer were followed strictly throughout the procedure. Briefly, ∼20 × 10^3^ cells per sample were loaded into the Chromium Controller and partitioned GEMs. Subsequently, the GEMs were lysed to release poly(A) mRNAs, the barcoded primers containing cell-specific barcodes, transcript-specific unique molecular identifier (UMI), and poly(dT) oligonucleotides for reverse transcription. Reverse transcription was then performed with the poly(A) mRNAs in an Applied Biosystems Proflex thermal cycler (Thermo Fisher Scientific, Waltham, MA) to generate cDNAs, which were pooled and cleaned of leftover reagents with Dynabeads MyOne silane beads (10x Genomics, Pleasanton, CA). The barcoded full-length cDNAs generated were amplified by PCR (12 cycles). The amplified cDNAs were purified with SPRIselect reagent (Beckman Coulter, Brea, CA), subjected to quality control, and quantified with the Agilent high-sensitivity D5000 kit (Agilent, Santa Clara, CA) on a TapeStation bioanalyzer (Agilent, Santa Clara, CA). The final reaction steps involved the addition of Illumina’s P5, P7, and Read2 primers and the sample index tag to the cDNA products. Briefly, the following steps were performed: enzymatic fragmentation, cDNA end repair and A-tailing, followed by double-tailed size selection with SPRIselect. Adaptor ligation, sample index PCR (16 cycles), and double-tailed size selection (with SPRIselect) were then performed. The libraries obtained were then subjected to quality control, quantified with the Agilent high-sensitivity D5000 kit on a TapeStation bioanalyzer, and sequenced on an Illumina NovaSeq 2 × 150 instrument (Illumina, San Diego, CA). FASTQ files of sequenced reads of three categories were generated: Read1 (28 bp, containing the 16-bp cell barcode and 12-bp UMI), index (8 bp, containing the sample index tags), and Read2 (91 bp, containing the transcripts).

### scRNA-seq bioinformatics analysis

scRNA-seq data analysis was performed with the single-cell analysis workflow of CLC Genomics Workbench v21 software (Qiagen, Venlo, the Netherlands), which includes an inbuilt Cell Ranger pipeline for scRNA-seq dataset preprocessing and downstream analysis. The dog (*Canis lupus familiaris*) genome (Ensembl ROS_Cfam_1.0; GCA_014441545.1) was incorporated into the workflow in place of the default human genome. The sequencing datasets for the four samples were imported, and the single-cell workflow was performed with the default parameters of Cell Ranger v7.0.0. The reads were demultiplexed to identify barcodes and to match each R2 read (transcript) to the corresponding R1 read (UMI and cell barcodes). The demultiplexed reads were trimmed based on quality score, nucleotide ambiguity, abnormal length, and homopolymer trimming, to exclude poor-quality reads. The retained reads were mapped against the *C. lupus familiaris* genome/transcriptome to identify the genes sequenced. This mapping step generated a gene expression matrix consisting of cells and the list of genes expressed within them, identified by means of the single-cell barcodes. Doublets containing more than one cell were also identified and excluded from subsequent analysis. Low-quality cells were removed, and background noise was limited by manual adjustment of the pipeline to remove all cells with <200 identified genes, as described in previous single-cell studies (18). Only a minimal number of doublets, representing ∼8% of the cells, were identified and subsequently excluded from further analysis (Table I). The filtered matrix was normalized, batch effect corrected, and merged into a single combined matrix for cluster generation and subsequent analysis. Cells were clustered by principal component analysis (20 dimensions) based on their gene expression profiles. The clusters were visualized as two-dimensional uniform manifold approximation and projection plots with a Leiden resolution (clustering parameter) of 0.3. Clusters were manually annotated to identify cell types based on differentially expressed genes and known canonical cell type–specific markers from the CellMarker database (https://bio-bigdata.hrbmu.edu.cn/CellMarker/), the PanglaoDB database (https://panglaodb.se/index.html), or as reported in previous studies. Downstream analysis was performed to identify cell subpopulations based on marker gene expression, leading to the creation of an immune cell atlas for the canine PPs.

Additionally, to perform pseudobulk scRNA-seq analysis, the scRNA-seq reads were pseudomapped to a reference transcriptome with kallisto software (19). Briefly, pseudomapping is a computational technique that assigns reads to transcript sequences based on their compatibility with the transcriptome (19). Next, the estimated read counts (est_counts) obtained for each transcript have been concatenated at the gene level. Estimated counts per gene were normalized using the DESeq2 package (20) in R software. DESeq2 normalization adjusts for differences in library size and composition, ensuring that the expression values are comparable across samples. The normalized counts were further analyzed and used for downstream analysis and visualization, on heatmaps for example.

### Statistical analysis

Statistical analyses were performed with GraphPad Prism version 9 (GraphPad). Differences in cell marker or gene expression were evaluated by two-way ANOVA and a Mann–Whitney *U* test for nonparametric comparisons. We used two biological replicates for flow cytometry and four for scRNA-seq analysis.

## Results

### Structural organization of canine PPs

**FIGURE 1.**
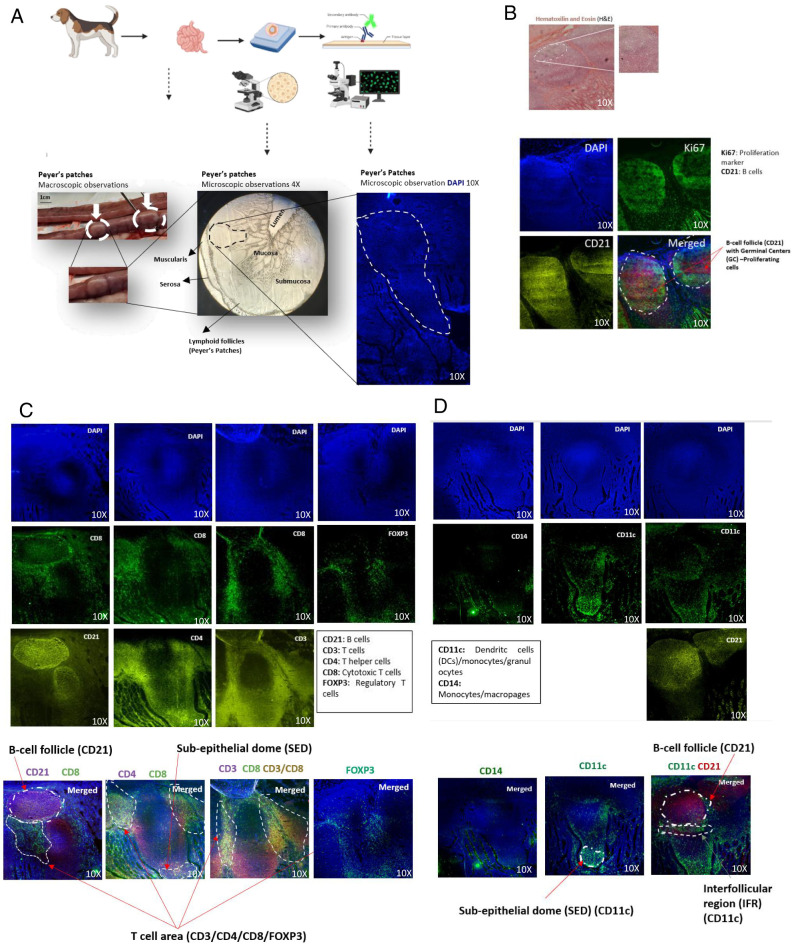
Structural organization of canine Peyer’s patches, according to immunofluorescence data. (**A**) Sample collection and preparation. Intestinal tissues from two dogs were sampled, and regions of ∼2 cm in length were identified as Peyer’s patches (PPs). The PPs were isolated, sectioned on a cryostat, and prepared for microscopy, with the various structures within the intestine identified before DAPI staining. (**B**) Visualization of PPs with H&E show different staining intensities within the follicles. The staining with Ki67, for proliferating cells, and CD21, for B cells, allows the visualization of proliferating B cells within the GC. (**C**) Immunofluorescence staining of PPs. PPs were stained with DAPI in combination with Abs against CD8 and one of the following markers: CD21, CD4, or FOXP3. Epifluorescence microscopy is shown at original magnification ×10, visualizing specific structures, including the subepithelial dome (SED), T cell areas, and B cell follicles. (**D**) Myeloid cell staining. Immunostaining of the CD14 and CD11c markers, along with CD21, was used to identify dendritic cells within the SED. Different images from the lower and upper parts of the sample were captured to adequately show the CD11c staining in proximity to both the epithelium and the follicle.

We first used immunofluorescence techniques to examine the PPs in the duodenum and jejunum of two dogs (Fig. 1A). Macroscopic examination revealed distinct white structures, ∼2 cm across, along the length of the intestines (Fig. 1A). Under the microscope, we were able to distinguish the mucosa, submucosa, and the lymphoid follicles present within the PPs (Fig. 1A). The canine PPs observed had the characteristic elongated shape (Fig. 1A), consistent with previous descriptions in the canine jejunum (13). H&E staining was lighter in the upper area (Fig. 1B), suggesting the presence of proliferating GC cells, as in other species (21). We characterized canine PPs, confirming phenotypic patterns observed in other species using specific immunostaining markers. CD21 staining revealed a distribution of B cells concentrated within the follicles (Fig. 1B), with each canine PP consisting of multiple consecutive follicles, each containing central areas densely populated with B cells. Staining with Ki-67, a proliferation marker, identified the B cells proliferating within the follicle, designating the dark zone of the GC (22). The presence of CD21^+^ B cells within this zone, along with the high concentration of proliferating cells, further confirmed it as the B cell follicle and indicated the presence of the GCs (Fig. 1B). Staining for CD3, CD4, and CD8 was used to identify mature T cells, including Th and cytotoxic T cells. With this immunostaining approach, we were able to identify most of the T cells in the areas surrounding the B cell follicles, and, to a lesser extent, in the SED of the PPs (Fig. 1C). CD4^+^ and CD8^+^ T cells had a similar distribution, but CD4^+^ T cells were more abundant than CD8^+^ T cells (Fig. 1C). Additionally, FOXP3 staining, used to identify regulatory T cells (Tregs), was also detected between the follicles (Fig. 1C). The T cell markers were distributed between follicles, designating the T cell areas of the PPs. For myeloid cells, CD11c staining was used to identify DCs and CD14 staining was used to detect macrophages. CD11c^+^ cells were located principally close to the epithelium and, to a lesser extent, adjacent to the follicles (Fig. 1D). Costaining for CD21 and CD11c demonstrated the presence of CD11c^+^ cells surrounding the B cells (Fig. 1D). However, no specific CD14 staining was detected within the PPs (Fig. 1D). The higher abundance of CD11c^+^ cells and of T cells close to the epithelium defined the location of the SED (Fig. 1E). Moreover, the presence of CD11c^+^ cells close to B cell follicles (Fig. 1D) suggested the presence of an interfollicular region, in which follicular DCs interact with T cells, facilitating Ag presentation and subsequent B cell activation (14).

These findings highlight the characteristic organization of B, T, and myeloid cells within canine PPs. B cell follicles serve as pivotal points for B cell activity, and the adjacent interfollicular and T cell areas house a considerable population of CD4^+^ and CD8^+^ T cells, with CD11c cells located close to the epithelium and next to the T and B cell zones. This arrangement suggests a dynamic interaction between these immune cell populations and provides important insight into the cellular composition and functional organization of PPs in dogs.

### Canine M cells in the gut FAE express GP2

**FIGURE 2.**
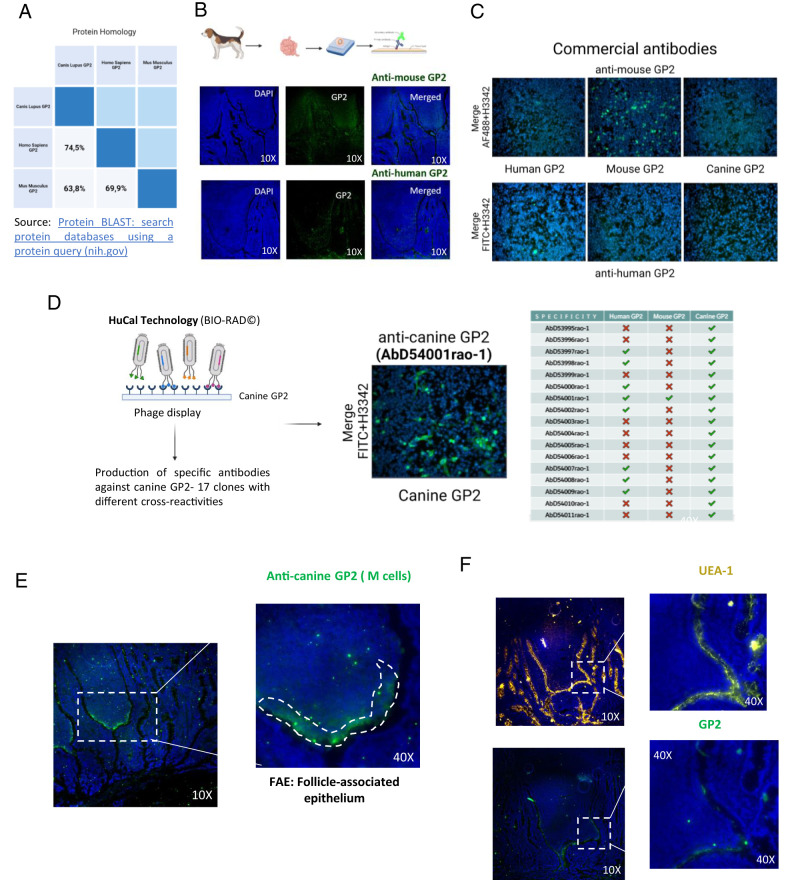
Identification of canine M cells with a specific anti-canine GP2 Ab. (**A**) Protein homology analysis. The sequence similarity of the *Canis lupus* (dog) and *Mus musculus* (mouse) or *Homo sapiens* (human) GP2 proteins was investigated. The canine sequence showed 74.5% similarity to the human sequence and 63.8% similarity to the mouse sequence. (**B**) Cross-reactivity assessment. Commercial Abs against human and mouse GP2 were tested for cross-reactivity with canine GP2 in tissue samples. No cross-reactivity was observed. (**C**) Transfected cells expressing canine, murine, and human GP2 were used to confirm the specificity of commercial Abs. The Abs tested specifically recognized GP2 from the corresponding species, but not from other species. (**D**) Ab production and validation. Bio-Rad HuCAL technology was used to generate 17 clones against canine GP2, which were validated with transfected cells. (**E**) Immunostaining for canine GP2. Canine Peyer’s patch sections were permeabilized and stained for canine GP2. Immunofluorescence imaging at original magnification ×10 and ×40 revealed specific staining in the follicle-associated epithelium region. (**F**) Specificity comparison. The same procedure, with UEA-1 staining, revealed nonspecific binding across the epithelium and mucus, contrasting with the specific staining observed with the Ab against canine GP2.

Next, we wanted to characterize the FAE, evaluating the presence and distribution of specialized M cells within the canine epithelium. In PPs, the FAE and M cells play a crucial role in Ag trafficking before DC processing (23). However, our investigations were limited by the absence of commercially available markers targeting canine GP2, a specific marker of M cells (7). We overcame this limitation by identifying commercial Abs against the human and mouse GP2 proteins. There is 74.5% amino acid sequence identity between the *Homo sapiens* and *C. lupus* GP2 proteins, and 63.8% sequence identity between the *Mus musculus* and *C. lupus* GP2 proteins (Fig. 2A). Despite the relatively low level of similarity between the proteins of the different species, we tested these Abs in canine PP tissue with the hope of observing cross-reactivity, but we detected no signal indicating binding to canine GP2 (Fig. 2B). We assessed the cross-reactivity of these commercial Abs further by transfecting cells with specific plasmids encoding for canine, murine, or human GP2 and the FLAG tag as a positive control (Fig. 2C). We then tried to detect the GP2 proteins with the commercial Abs in positively transfected cells. The anti-human GP2 Ab weakly detected human GP2 but it did not recognize canine or murine GP2 (Fig. 2C). Similarly, the anti-mouse GP2 Ab strongly detected murine GP2 but did not recognize human or canine GP2 (Fig. 2C). The expression of the three proteins was confirmed by detecting the FLAG epitope, which was encoded at the C terminus of each protein, using a FLAG-specific Ab.

We addressed the need for a specific Ab targeting canine GP2 by commissioning, from Bio-Rad, the production of such an Ab using HuCAL technology, as previously described in this study. As a result, 17 Fab clones were obtained against partial canine GP2. In vitro testing using BHK21 transfected with recombinant plasmid encoding for canine GP2-FLAG protein confirmed that all of these Abs successfully detected the canine GP2 protein in transfected cells (Fig. 2D). Among the 17 clones, 8 also recognized human GP2 (47%), and 1 of these 8 clones also detected murine GP2 (5.8%) (Fig. 2D). Hence, this anti-canine GP2 Abs demonstrated higher rates of cross-reaction with human than with mouse GP2. These differences in cross-reactivity can be attributed to the low identity in the region of the expressed protein used for panning. Following the validation of the Abs in transfected cells, we then assessed their ability to detect M cells specifically in cryosections of canine PPs. Two different clones highly specific to canine GP2 (GP2_004) or cross-reactive in vitro with the proteins of all three species (GP2_001) were tested, with no striking difference in the results obtained (Supplemental Fig. 1). Specific staining of GP2 was clearly observed in the FAE of canine PPs, predominantly in areas close to the intestinal lumen (Fig. 2E). Moreover, upon manual visual counting of the epithelial cells on the apical side, the frequency of M cells appears to be ∼30% of the total epithelial cells specifically located in the FAE lining the lumen. This observation is consistent with the anticipated Ag-trafficking process occurring within the intestinal lumen (23). CD11c^+^ cells were highly abundant adjacent to the localized M cells, consistent with an interaction between M cells and DCs in immune responses.

*Ulex europaeus* agglutinin-1 (UEA-1), a lectin that binds to α(1,2)fucose, is commonly used to identify M cells in mice or humans but can also recognize goblet cells (24). We assessed the specificity of this lectin for the detection of canine M cells once we had a validated anti-canine GP2 Ab for comparison. We found that UEA-1 staining was not specific to M cells. In contrast to the specific anti-GP2 Ab, the staining achieved with this lectin extended throughout the entire epithelium and beyond (Fig. 2F). In conclusion, we have successfully validated an anti-GP2 Ab specific to canine M cells, both in vitro and in tissue. This Ab will be a valuable tool for studying canine PPs, as lectins, such as UEA-1, which is used for this purpose in other species, yield nonspecific staining in canine PPs.

### Higher regulatory profile of double-positive CD4^+^CD8a^+^ T cells in canine PPs

**FIGURE 3.**
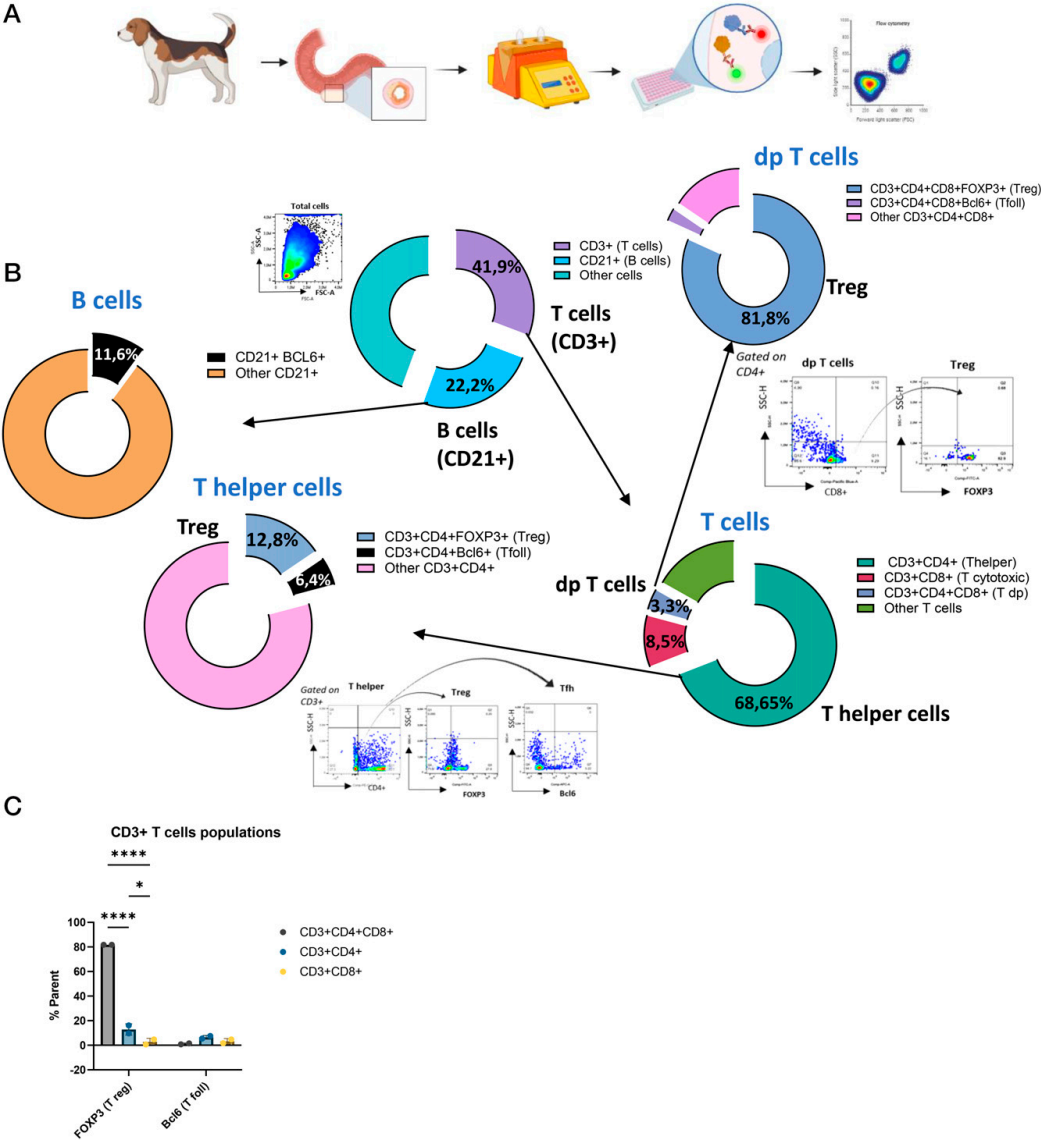
Characterization of T and B cell populations in canine Peyer’s patches by flow cytometry. (**A**) Peyer’s patches (PPs) from two dogs were dissociated, and single-cell suspensions were stained for flow cytometry in a Cytek Northern Lights flow cytometer. (**B**) Proportions of T and B cells. T cells accounted for 41.9% and B cells for 22.2% of the cell population analyzed. Within the B cell population, 11.6% of the cells were positive for Bcl-6. T cells were further separated into CD4^+^, CD8^+^, and CD4^+^CD8^+^ subsets, revealing high levels of heterogeneity in the expression of the regulatory marker FOXP3. (**C**) FOXP3 expression in T cell subsets. The percentage of FOXP3^+^ cells was significantly higher among CD3^+^CD4^+^CD8^+^ cells than among CD3^+^CD4^+^ or CD3^+^CD8^+^ cells. Statistical analysis was performed by two-way ANOVA. * *p* ≤ 0.05, ****p ≤ 0.001. dp, double-positive.

The cells of PPs from two dogs were dissociated and stained for specific immune cell markers (Fig. 3A), such as Bcl-6 and FOXP3 transcription factors, which can be used to identify follicular and regulatory cells, respectively (Supplemental Fig. 2). Immunofluorescence analyses demonstrated the presence of B and T cells and their subtypes but could not determine their relative proportions. However, flow cytometry made it possible to determine these proportions and to investigate other cell markers. An analysis of the total population of stained cells indicated that T cells accounted for 41.9% of the total cell population, whereas B cells represented 22.2% (Fig. 3B), with 11.6% of B cells positive for Bcl-6, suggesting the presence of GC B cells (25). We also characterized T cell subtypes: 68.65% were identified as CD3^+^CD4^+^ Th cells, 8.5% as CD3^+^CD8a^+^ cytotoxic T cells, and 3.3% as CD3^+^CD4^+^CD8a^+^ double-positive T cells. These double-positive cells may correspond to those previously reported to be present in the secondary lymphoid organs of healthy dogs (26). We found that 12.8% of Th cells had a Treg phenotype and 6.4% were positive for Bcl-6, suggesting the presence of regulatory and follicular T cells (Fig. 3B). Remarkably, the vast majority (81.8%) of double-positive T cells were found to be Tregs (Fig. 3B). Moreover, significant differences in phenotypic profile were observed between different T cell subtypes, particularly with respect to the expression of FOXP3, which was significantly stronger in CD3^+^CD4^+^CD8a^+^ cells than in CD3^+^CD4^+^ and CD3^+^CD8a^+^ cells (Fig. 3C). These findings highlight the intriguing regulatory phenotype of double-positive T cells in canine PPs and shed light on the presence of follicular cell populations within canine secondary lymphoid tissues.

### Exploring cell populations in depth by scRNA-seq

**FIGURE 4.**
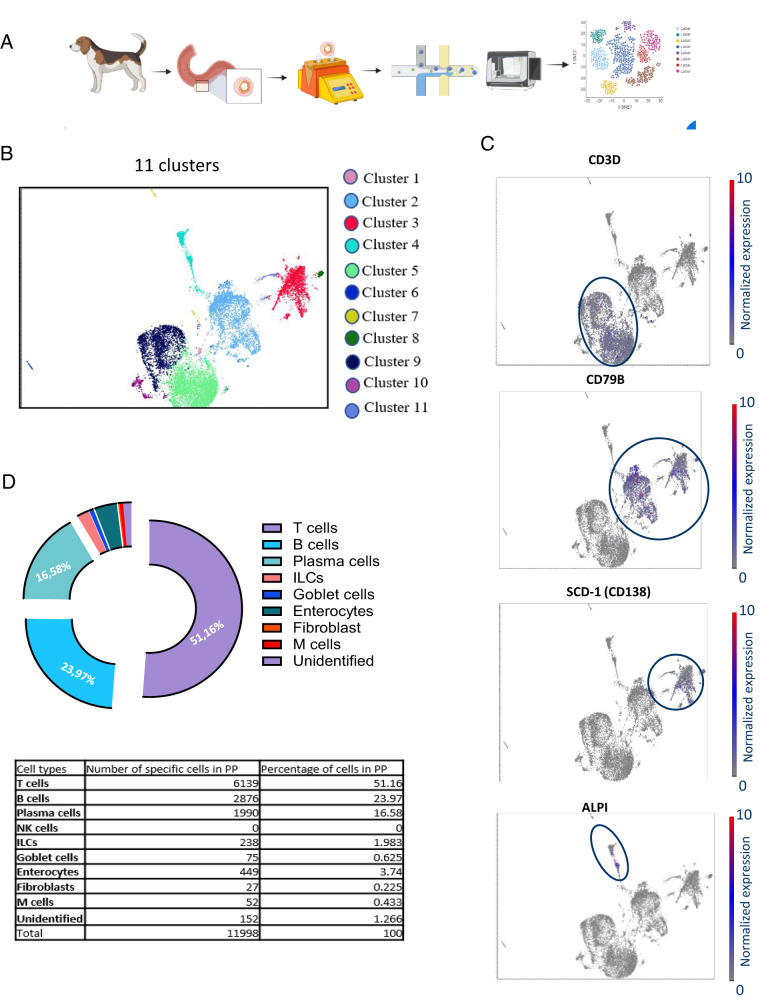
Cluster identification and cell type classification by scRNA-seq. (**A**) Peyer’s patches from four dogs were isolated, dissociated, and introduced into the microfluidic system for scRNA-seq analysis. (**B**) Cluster identification. Eleven clusters were identified with a Leiden resolution of 3.0, making it possible to classify different cell populations. (**C**) Marker expression. The expression of markers such as CD3D, CD79B, SCD-1, and ALPI was visualized across the identified clusters, facilitating cell type identification. (**D**) Cell populations identified. Nine cell populations were identified on the basis of gene expression profile. T cells, B cells, and plasma cells predominated.

We used scRNA-seq to deepen our understanding and to identify the subpopulations within the complex structures of the dog immune system. PPs were harvested from four dogs in steady-state conditions, and ∼20,000 cells per animal were processed and added to the microfluidic system for library preparation and sequencing (Fig. 4A). Individual cells were clustered on the basis of similarities in their transcriptional profiles, and the resulting cell clusters were visualized on a two-dimensional uniform manifold approximation and projection plot. With a Leiden resolution of 0.3, we identified 11 distinct clusters (Fig. 4B) with a total of 11,998 cells and 14,365 unique genes (Table I). We then annotated the specific cell types within each cluster manually based on the identification of known canonical cell type–specific markers (Fig. 4C). We also annotated seven known cell types: T cells, B cells, plasma cells, enterocytes, innate lymphoid cells (ILCs), M cells, and goblet cells (Fig. 4D). B cells were identified based on their expression of CD19, CD20, CD79A, and CD79B; they accounted for ∼23.97% of the total number of annotated cells (Fig. 4C). T cells were identified on the basis of their expression of *CD3D*/*CD3E* (Fig. 4C) and *LCK* (lymphocyte-specific protein tyrosine kinase); they accounted for ∼51.16% of the cells. Plasma cells were identified on the basis of their expression of B cell markers and *SDC-1* (CD138) (Fig. 4C) (27, 28). NK cells were identified on the basis of their expression of *NKG7* (NK cell granule protein 7), *GZM* (granzyme), *PRF1* (perforin 1), *CD2*, and *CD160* (27, 28). Epithelial cells were identified on the basis of their expression of epithelial cell adhesion molecule (*EpCAM*) (29). Fibroblasts were identified on the basis of their expression of *PDGFRA* (platelet-derived growth factor receptor α) (29). Enterocytes were identified on the basis of their expression of *ALPI* (alkaline phosphatase, intestinal) (Fig. 4C), *SLC26A6* (solute carrier family 26 member 6), and *FABP2* (fatty acid-binding protein 2) (18), whereas goblet cells were identified on the basis of their expression of *TFF3* (trefoil factor 3), *SPINK4* (serine peptidase inhibitor Kazal type 4), and *CLCA1* (chloride channel accessory 1) (18). Finally, M cells were identified on the basis of their expression of zymogen granule membrane protein-2 (GP-2) and ILCs were identified as CD45^+^ cells (expressing protein tyrosine phosphatase, receptor type, C [*PTPRC*]) lacking the expression of distinct T cell, B cell, or other immune cell lineage-specific markers but with specific expression of *IL7R* (CD127) (30). Approximately 99% of the cells were successfully annotated, but no myeloid cells were captured in the dataset. This may reflect losses during the filtering process or low levels of canonical marker expression; the small proportion of unidentified cells (∼1%) in the dataset may correspond to myeloid cells. Nonimmune cells, probably from the epithelial and stromal layers (M cells, goblet cells, enterocytes, fibroblasts, and epithelial cells), and unidentified cells were removed and excluded from downstream analyses to enable us to focus on the identified immune cells. Our scRNA-seq analysis made it possible to annotate and characterize the immune cell populations within canine PPs comprehensively, providing valuable insight into the cellular composition and heterogeneity of this important immune tissue.

**Table I.** Gene and cell counts and numbers of cells before and after filtering

Samples	Estimated No. of Cells	Median No. of Reads per Cell	Median No. of Genes per Cell	No. of Cells Passing Filter (>200 genes)	Identified Doublets	Retained Cells
PP1	7,068	288	171	3,013	336	2,677
PP2	8,902	251	188	4,038	777	3,261
PP3	8,395	277	173	3,468	630	2,838
PP4	8,290	249	184	3,743	521	3,222
Total	32,655			14,262	2,264	11,998

The Estimated No. of Cells is the initial number of cells detected, whereas Retained Cells indicates the number of cells after the elimination of cells expressing 200 identified genes and the removal of doublets. PP, Peyer’s patch.

### Analysis of cell subpopulations in canine PPs

**FIGURE 5.**
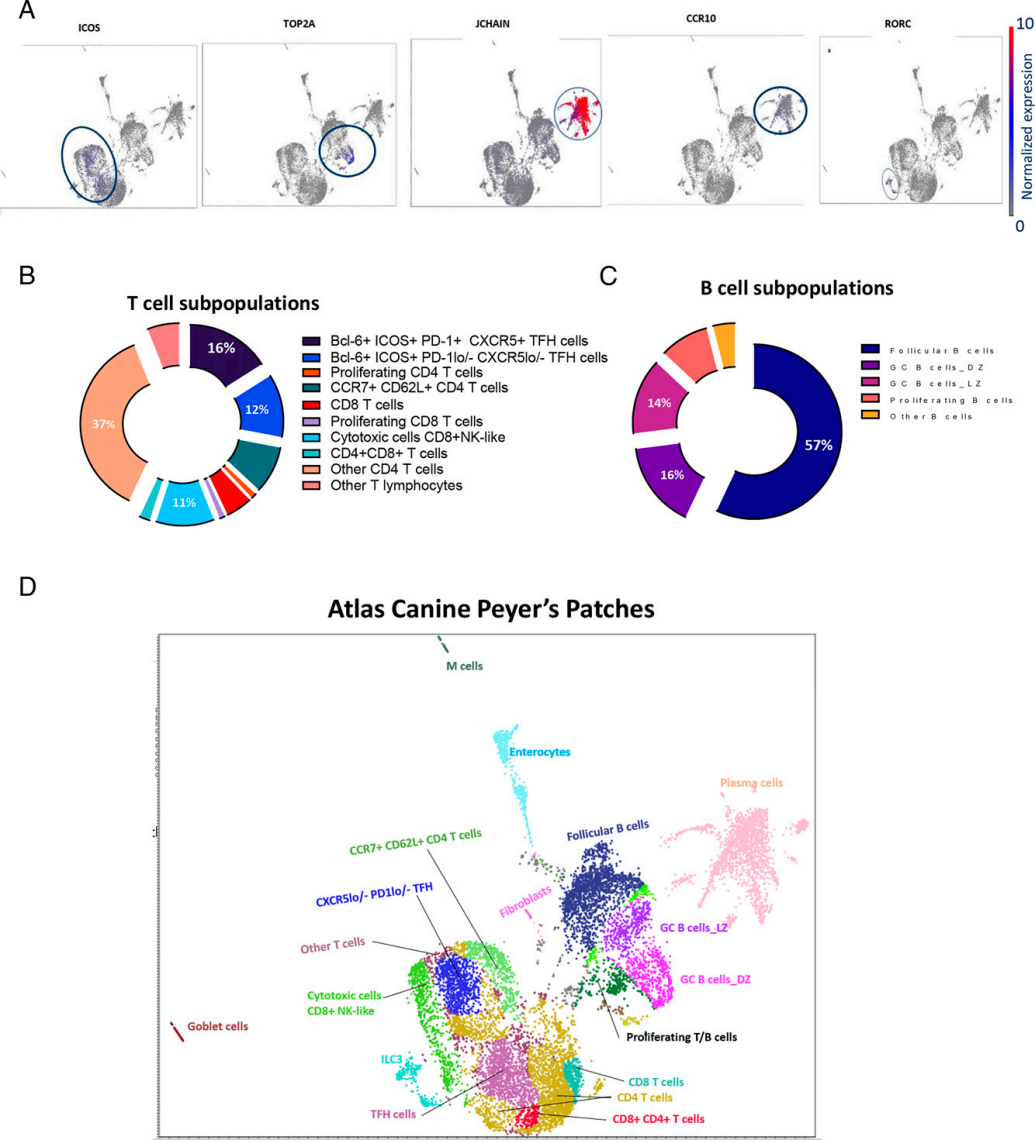
Gene expression distribution, B and T cell subpopulations, and the Peyer’s patch atlas. (**A**) Marker expression. The expression of markers such as ICOS, TOP2A, JCHAIN, CCR10, and RORC was visualized across the clusters, facilitating cell subtype identification. (**B**) T cell subpopulations. The distribution of different T cell subpopulations is presented as a pie chart, with the corresponding percentages. (**C**) B cell subpopulations. The distribution of different B cell subpopulations is shown as a pie chart, with the corresponding percentages. (**D**) We propose an annotation of the immune cells identified within clusters, based on the specific expression patterns of marker genes. Bcl-6, B cell lymphoma 6; CD62L, CD62 L-selectin; DZ, dark zone; GC, germinal center; ILC3, innate lymphoid cells group 3; LZ, light zone; PD-1, programmed cell death protein 1; Tfh, T follicular helper cell.

We explored the diversity within the immune clusters identified further by conducting a detailed subpopulation analysis for each annotated cell type. T cells were initially identified on the basis of CD3 expression and constituted the largest population of cells, accounting for ∼51% of all cells in PPs. We identified 6139 T cells in total. The largest subpopulation was CD4^+^ T cells, which accounted for 64% of the total T cell population, whereas CD8^+^ T cells accounted for 17% of the total CD3^+^ cell population (Fig. 5B). A small cluster of cells expressed both *CD4* and *CD8A*, as observed in flow cytometry analysis, and were putatively annotated as CD4^+^CD8^+^ T cells; these cells accounted for 2% of the total T cell population. The expression of the *CD4* and *CD8A* T cell markers was unclear for other CD3^+^ cells, accounting for ∼6% of the cell population. These cells may have been γδ T or NKT cells, but they lacked the known canonical lineage-specific markers for γδ T cells and NKT cells. Within the CD4^+^ T cell population, T follicular helper (Tfh) cells, expressing the lineage marker *ICOS* (Fig. 5A), *PD-1*, and/or *CXCR5* (Fig. 5B), accounted for 28% of the total T cell population. These cells also clearly expressed Bcl-6, a transcription factor that commits CD4 T cells to the Tfh phenotype (25). Two Tfh phenotypes were observed: Bcl-6^+^ICOS^+^PD-1^+^CXCR5^+^ Tfh cells (16%) and Bcl-6^+^ICOS^+^PD-1^lo/−^CXCR5^lo/−^ Tfh cells (12%) (Fig. 5B). In addition to the CCR7^+^CD62L^+^ central memory CD4 T cell population (9%), a small population of proliferating CD4 T cells was present (1%); other non-Tfh and nonproliferating CD4^+^ T cells accounted for 37% of the total T cell population (Fig. 5B). We identified two distinct subpopulations in addition to canonical CD8^+^ T cells. The first subpopulation consisted of proliferating CD8 T cells (1%), characterized by the expression of cell cycle/proliferation markers, and labeled as proliferating CD8^+^ T cells (Fig. 5B). The second subpopulation was remarkable, comprising CD3^+^CD8^+^ T cells expressing NK cell markers, including *CD2*, *CD160*, *GZM*, and *PRF-1*. Given their coexpression of CD8A T cell markers, these cells were classified as “cytotoxic cells” rather than NK cells (31) (Fig. 5B). We therefore made no separate annotation for NK cells, as the characteristics of these cells overlapped with those of the cytotoxic cell population.

B cells were initially identified on the basis of their *CD19*, *CD20*, *CD79A*, and/or *CD79B* expression. The subpopulations of B cells were divided into four subtypes (Fig. 5C). One subpopulation of B cells was found to be FCER2^+^ (encoding CD23) CXCR5^+^ and these cells were putatively annotated as follicular B cells (32). A second subpopulation of B cells expressed AICDA (activation-induced cytidine deaminase), a marker of cells potentially undergoing affinity maturation (33). Some of these cells expressed proliferation markers (*TOP2A* and *PCLAF*) (Fig. 5A) together with AICDA, indicative of proliferating GC cells, and these cells were defined as dark-zone GC B cells (22). The other AICDA^+^ cells did not express proliferation markers and were classified as GC light-zone B cells because they expressed *AICDA* and *CXCR5*, indicating that they were nonproliferating GC cells potentially interacting with Tfh cells in the GC light zone (22). A third population of B cells consisted of proliferating B cells expressing proliferation markers (without *AICDA* and *CXCR5*). Thus, 57% of the 6139 B cells identified were follicular B cells, 16% were dark-zone GC B cells, 14% were light-zone GC B cells, and 8.5% were proliferating B cells (Fig. 5C). Plasma cells were initially identified on the basis of their expression of B cell markers together with CD138, a lineage-specific marker for plasma cells. These cells accounted for ∼17% of cells identified in PPs (Fig. 4B). The subtype specificity of plasma cells could have been determined according to the Ig subclass genes expressed. However, the dog genome used in this study did not include Ig annotations, rendering the direct interpretation of plasma cell subpopulations more difficult. Nevertheless, the plasma cells in this study displayed very high levels of *JCHAIN* (encoding Ig joining chain) expression (Fig. 5A), suggesting that they were multimeric Ig-secreting plasma cells, and therefore either IgM^+^ or IgA^+^ plasma cells. These cells were also CCR10^+^ (Fig. 5A), consistent with cells of the IgA^+^ subtype that eventually home to the mucosa to secrete IgA. As CCR10 is a gut-homing receptor, the Ig present was almost exclusively of the IgA subtype and the plasma cells were IgA^+^ (34). Finally, ILCs were initially identified as CD127^+^ cells lacking the lineage-specific markers defining other cell types. They accounted for 2% of the total cell population in the PPs analyzed (Fig. 4D). Further investigation revealed that they expressed *RORC* (retinoic acid receptor–related orphan receptor C) (Fig. 5A), *RORA*, and *IL-22*, suggesting they were probably of the ILC3 subtype, given their expression of these Th17-related genes (30).

Finally, we developed a marker-guided immune cell atlas based on the cell populations and subpopulations identified. This atlas shows the immune cell types and subtypes present at baseline in the PPs of healthy dogs (Fig. 5D).

### Exploring myeloid gene expression through single-cell pseudobulk analysis

**FIGURE 6.**
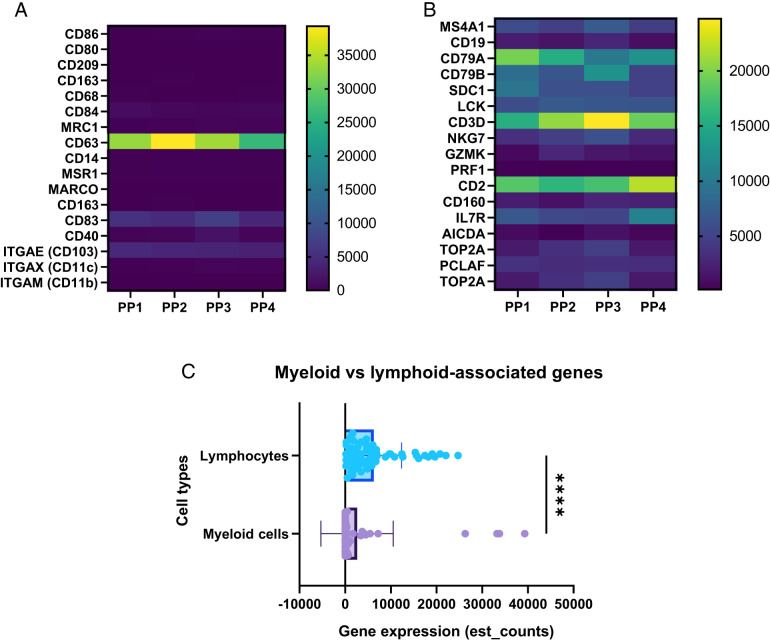
Comparative pseudobulk analysis of scRNA-seq data: myeloid-associated versus lymphoid-associated genes. (**A**) Heatmap representation of 17 representative myeloid genes identified by pseudobulk scRNA-seq analysis. (**B**) Heatmap representation of 17 representative lymphoid genes identified by pseudobulk scRNA-seq analysis. (**C**) Comparison of gene expression (est_counts) for the selected genes between myeloid and lymphoid cells, revealing a significant difference. A Mann–Whitney *U* test was used for statistical analysis. ****p ≤ <0.0001.

We have successfully generated an atlas of the major subpopulations present in canine PPs based on scRNA-seq analysis. However, no myeloid cells were detected in our analysis, despite the confirmation of the presence of myeloid markers, such as CD11c, in our immunofluorescence analysis (Fig. 1D), and the identification of myeloid cells in PPs by alternative techniques in previous studies (35). We therefore decided to explore the raw scRNA-seq data before filtering to try to determine why myeloid cell markers were not present in our final dataset. We pseudomapped the raw sequence reads obtained from the scRNA-seq data with Kallisto (19) and normalized the estimated counts using the DESeq2 package and R software to obtain pseudobulk scRNA-seq results. With this approach, we were able to analyze gene expression at the sample rather than single-cell level, as for bulk RNA-seq. We further investigated the reasons for which myeloid cell genes were not detected in the scRNA-seq data through a comparison with the highly expressed lymphoid cell genes identified in the scRNA-seq analysis. As shown in the heatmap (Fig. 6A), the levels of canonical myeloid gene expression were lower than the levels of lymphoid gene expression (Fig. 6B). In parallel, we compared the expression levels of the top 17 myeloid genes with those of the top 17 lymphoid genes. Expression levels were significantly higher for the lymphoid genes (Fig. 6C). These findings suggest a differential pattern of expression between myeloid cells and lymphocytes, with lymphoid cells displaying higher levels of expression for the genes analyzed. This observation suggests that the annotation of myeloid genes could be used to identify innate immune cell populations in dogs. However, due to the low levels of expression of myeloid genes observed at the bulk scRNA-seq level, myeloid cells may not meet the filtering criteria during the downstream analysis of scRNA-seq data.

## Discussion

An understanding of the cellular architecture of the GALT will pave the way for a detailed understanding of the underlying immune mechanisms at steady state. PPs play a crucial role in initiating mucosal immune responses and facilitating IgA secretion (36). An understanding of their characteristics is, therefore, important for the development of novel mucosal vaccines and the analysis of various diseases, where the dog is a valuable model (37). Much is known about these structures in humans and mice, but much less is known about the PPs of dogs (13). The challenge of studying canine PPs has been rendered even greater by the inability of many existing human and mouse Abs to bind to the corresponding protein in dogs, making it difficult to delineate canine immune cellular phenotypes. To address these gaps, we used a scRNA-seq platform to characterize the immune architecture of healthy dog PPs, marking (to our knowledge) the first study of its kind. Furthermore, our successful validation of anti-canine GP2 Abs allowed the identification of canine M cells for (to our knowledge) the first time.

We first classified the PP regions described in other species (38) for dogs, including the B cell follicles with GCs, crucial for affinity maturation after B cell activation by follicular T cells (6), the T cells adjacent to the follicles, and the SED rich in DCs for Ag uptake and processing. However, although immunofluorescence is a very valuable technique for visualizing the structure and organization of tissues, it has several drawbacks. For example, it is difficult to observe more than two targets simultaneously due to the limited number of fluorophores available, and there are few available specific markers and Abs for use in dogs.

We then aimed to delineate the FAE and to identify the M cells present in this tissue. M cells are specialized cells present in the FAE of PPs that play a crucial role in Ag trafficking and IgA responses (14). M cells have been described in several species, including humans (39), mice (39), rats (40), pigs (41), horses (42), chickens (43), and cats (44), in various mucosal sites (45). However, the identification of M cells has presented challenges due to variations in markers used for detection, influenced by differences in carbohydrate expression among species (45). Cats, for instance, lack a specific marker for M cells and rely solely on structural characteristics identified by electron microscopy in the conjunctiva-associated lymphoid tissue (44). Meanwhile, in humans and mice, GP2 has been identified as a consistent universal marker for M cells (39). In dogs, no specific marker is available for canine M cells and commercial ones do not cross-react. Therefore, Abs targeting canine GP2 were developed and validated that successfully identify canine M cells, providing a valuable tool that enables a cost-effective and efficient technique for M cell identification in dogs. With this Ab, we observed a homogeneous distribution of canine M cells along the apical dome of the FAE, contrasting with the absence of M cells in the dome apex of rabbits revealed with the use of vimentin as a marker (17), highlighting the species-specific differences between species.

The presence of M cells in dogs opens new avenues for research investigations in this field. For example, the in vivo ligated loop method (39), which involves directly injecting preparations into PPs ligated on both sides, can provide valuable insights into transcytosis across the epithelium. Similarly, several in vitro models have been developed for studying M cells that could also provide valuable information about vaccine trafficking (38). However, it is essential to carefully evaluate all of these models in dogs to assess their feasibility accurately. Such studies will enhance our understanding of the mechanisms involved in mucosal immune responses and could help to optimize delivery strategies for dogs. Given the unique abilities of M cells to sample Ags, targeting these cells with vaccines has been investigated in several species (46). Nevertheless, further research is necessary to confirm that targeting M cells in dogs could become a potential reality.

We also used flow cytometry to explore additional cell populations within the PPs. We investigated T cell subtypes and the presence of follicular and regulatory cells, which are essential for GC responses (47). To characterize Tfh cells, we only used Bcl-6; however, to obtain more comprehensive results, additional markers, such as *PD-1*, *ICOS*, *CXCR5*, and *GL7*, would be beneficial (47). Although the proportion of CD4^+^ Th cells was visibly greater than the proportions of other T cell subsets on immunofluorescence, flow cytometry provided us with the precise proportions (CD8/CD4 ratio of 1:8) and allowed us to identify both follicular and regulatory Th cells. Additionally, double-positive CD4^+^CD8^+^ T cells have been described as a heterogeneous population with regulatory and cytotoxic functions in dogs (26). However, our research revealed >80% of the double-positive T cells expressed FOXP3, indicating a predominantly regulatory profile. Similar double-positive T cells have also been identified in porcine PBMCs, displaying a memory profile (48), and in the intestinal LP of macaques, demonstrating an activated memory phenotype (49). To gain a deeper understanding of these cell populations and their functions in dogs and other species, further studies are warranted.

As a means of obtaining a deeper understanding of the populations described and identifying additional subpopulations not accessible with the available canine markers, we performed scRNA-seq on canine PPs. This unbiased and high-resolution technique allowed us to identify and characterize five main immune cell types: T cells, B cells, plasma cells, NK-like cells, and ILCs. Observations at the gene level provide hints about what is happening at the protein level, but they do not provide conclusive evidence of translation to generate the protein. However, we found a marked concordance between gene expression profiles and protein detection by flow cytometry for the identification of T and B cells, including Th cells. The agreement between gene and protein results provides validation for the scRNA-seq approach.

The PPs were densely populated with CD4 T cells and CD8 T cells. Tfh cells play important roles in GC reactions and humoral responses, specifically on affinity and long-term Ab responses (50) They express *CXCR5*, which facilitates trafficking to the B cell follicle in response to CXCL13, ICOS, and Bcl-6, which are crucial for Tfh cell differentiation and maintenance (22). They also express PD-1, which promotes the localization of Tfh cells in the GC region to preserve the rigor of the affinity selection that occurs in that zone (51). We observed a subpopulation of Tfh cells that were CXCR5^+^ICOS^+^Bcl-6^+^PD-1^+^, consistent with the findings of Madissoon et al. (33), who reported the presence of CXCR5^+^ICOS^+^PD-1^+^ Tfh cells in the human spleen (another secondary lymphoid organ) in scRNA-seq studies. Additionally, we observed varying levels of expression of *PD-1* and *CXCR5* within Tfh cells, which could correlate with their localization and compartmentalization within mucosal tissues, as suggested for human tonsils using multiplexed imaging (52).

This study was subject to several limitations. scRNA-seq is a powerful technique for describing and discovering new cell populations, but the lack of specific cell enrichment limited our ability to explore rare cell populations in depth. Myeloid populations were not identified, with B and T cells accounting for 74% of all of the cells present. Moreover, median gene/cell counts were low due to an amplification bias, with UMI duplicates. Another limitation of this study relates to the use of the Chromium 10x Genomics platform, based on microfluidic capture. This system is the fastest and most cost-effective, but it provides little control over the specific cells captured, potentially leading to a loss of rare cell types (53). This amplification bias may also have masked weakly expressed genes, some of which may be marker genes for specific cell types. These genes would therefore be either lost or poorly represented in our dataset, precluding identification of the associated cell populations. Finally, this study was also limited by the limited information about canine-specific cell markers for gene annotation.

Previous canine scRNA-seq studies encountered similar limitations, particularly as concerns canine gene annotation within the database, resulting in low confidence for the mapping of reads (54). Furthermore, a study analyzing canine bronchoalveolar lavage fluid failed to detect innate cells, such as macrophages. All but one of the myeloid-associated genes associated with these cell populations (with the exception being *MARCO*) remained undetected (54). Similarly, another study exploring the markers of canine atopic dermatitis failed to detect mast cells and eosinophils, which play a key role in this disease (55). It was suggested that the lack of detection was due the higher RNase levels, lower RNA content, and higher sensitivity to low temperatures of these cell types (55). Furthermore, certain human cell types have been shown to harbor genes that are easier to identify by bulk RNA-seq than by pseudobulk scRNA-seq, such as genes sensitive to dissociation and temperature (33). For instance, gene expression levels are lower on pseudobulk scRNA-seq for splenic macrophages (33). We performed pseudobulk scRNA-seq analysis on our raw data to assess the presence of myeloid genes before application of the 200-gene filter. We detected myeloid gene expression, but at a significantly lower level than lymphoid gene expression. Thus, despite the power of scRNA-seq, well-established protocols are required to ensure the recovery of rare and sensitive populations during the final analysis.

This study generated a highly informative map, providing a detailed overview of the immune cells in PPs. It also generated considerable information about the spatial distribution of cells, protein levels (flow cytometry), and gene expression profiles (scRNA-seq). Future studies will need to target myeloid cells specifically to determine the populations and subpopulations of myeloid cells present in the canine GALT, as DCs and monocytes/macrophages are required for the induction of immune responses in these tissues (56). One possibility would be the FACS labeling and sorting of DCs, monocytes/macrophages, and other myeloid cells, followed by the profiling of these sorted cells in the microfluidic system. Moreover, this baseline transcriptomic profiling of cell types would provide a comprehensive basis for the use of scRNA-seq in other conditions, such as intestinal diseases. Future scRNA-seq studies in mucosal vaccination could explore transcriptomic changes in GALT immune cells correlated with durable or poor vaccine responses, aiding in outcome prediction. Similarly, there is significant interest in exploring the modifications in GALT immune cells and their transcriptomic profiles to enhance the prediction of outcomes in dogs with intestinal diseases or cancer (57). This investigation is crucial due to the significant role of dogs as a model for human diseases, given the shared naturally occurring pathologies between the two species, offering valuable insights for human health (38).

## Supplementary Material

Supplemental Table 1 (PDF)
