# Supplementary material for: Characterization of Canine Peyer’s Patches by Multidimensional Analysis: Insights from Immunofluorescence, Flow Cytometry, and Single-Cell RNA Sequencing
Source: Immunohorizons. 2023 Nov 28;7(11):788–805. doi: 10.4049/immunohorizons.2300091 (PMC10696420; doi:10.4049/immunohorizons.2300091)
Supplement: Supplemental Table 1 (PDF) [file IH_2300091_Supplemental_1.pdf]

Name	Catalog number	Batch lot	Expiry date
10X Chromium Next GEM single cell 3' GEM kit v3.1 16 rxns	PN-1000123	200220	05/04/2022
10X Chromium Next GEM single cell 3' Library kit v3.1 16 rxns	PN-1000157	200202	30/04/2022
10X Chromium Next GEM single cell 3' Gel Bead kit v3.1 16 rxns	PN-1000122	162397	19/04/2023
10X Dynabeads MyOne SILANE	PN-2000048	162086	30/09/2023
10X Chromium Next GEM Chip G Single Cell kit 16 rxns	PN-1000127	162464	10/05/2022
10X Single Index Kit T Set A, 96 rxns	PN-1000123	162835	29/03/2023
Beckman Coulter SPRIselect reagenst	B23318	19221700	01/05/2023
Qiagen Buffer EB	19086	169036752	Not defined
Bio-Rad 10% Tween 20	1662404	64352139	21/01/2025
Invitrogen TE buffer	12090-015	2275016	29/04/2021
Merck Ethanol	1045000438 (BI storage)	K522524486	Not defined
Agilent High Sensitivity D5000 Reagents	5067-5593	0006587868	06/04/2022
Agilent High Sensitivity D5000 ScreenTape	5067-5592	0202216-364	15/02/2022

Supplementary Table 1: scRNA-seq library preparation reagents and/or kit

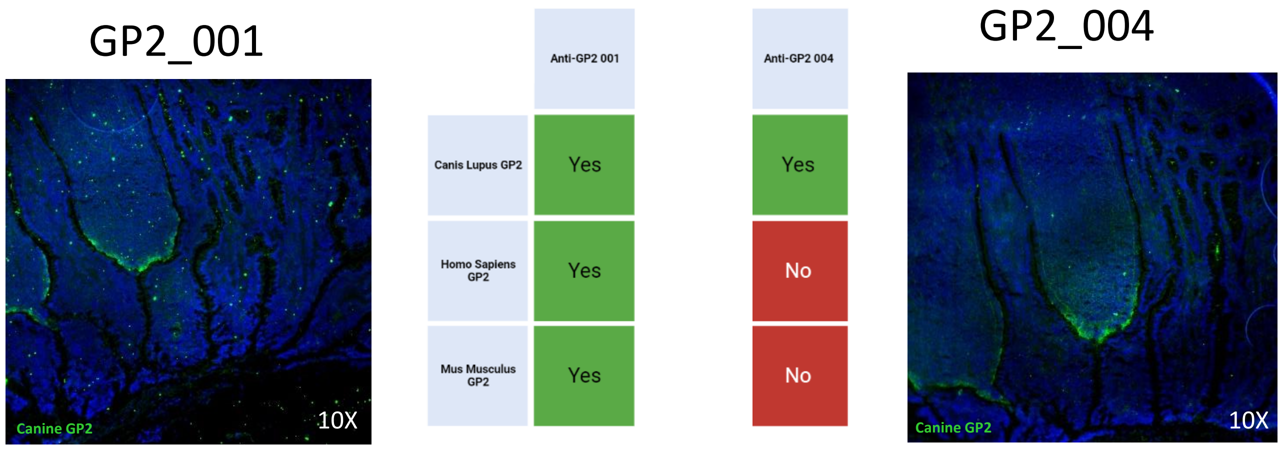

Supplementary Figure 1: Staining of Peyer's Patches with different anti-canine GP2 clones is comparable. Peyer's patches were fixed, permeabilized and stained with the clones of different specificities seen in transfected cells with GP2. GP2_001: cross-reacting between human, mouse and canine or GP2_004 only specific for canine GP2. The fluorescence was observed and compared between each staining at 10X.

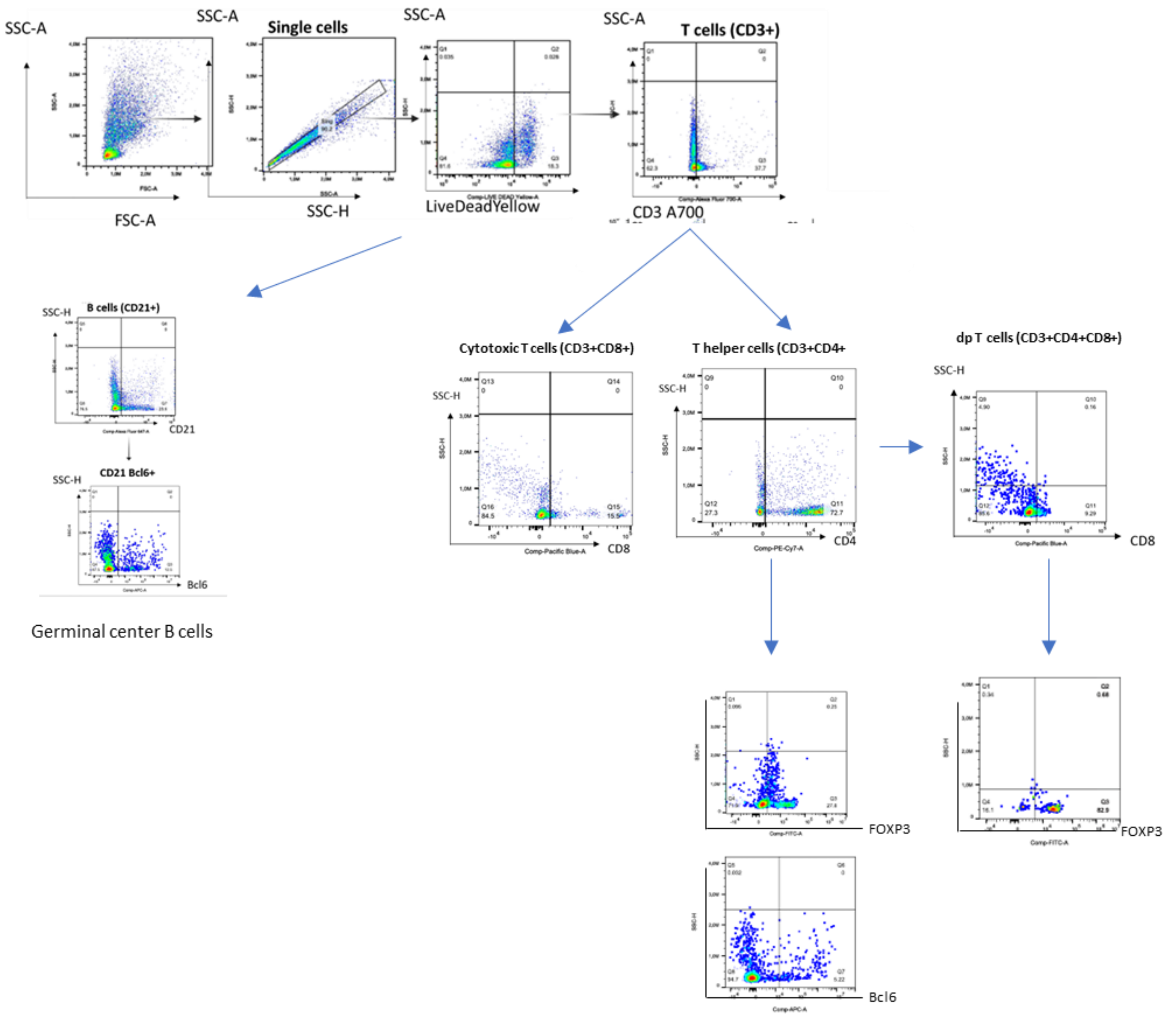

Supplementary Figure 2: Gating strategy of canine Peyer's Patches by flow cytometry. First, single cells were selected, followed by the viable cells. Then, cells were gated either with CD21 to identify B cells or CD3 to identify T cells. Bcl6 was explored in CD21. For the T cells, cytotoxic (CD3+CD8+) and Thelper (CD3+CD4+) were separated and FOXP3+, regulatory and Bcl6+, follicular, profiles were investigated.
